# Effectiveness of de-implementation strategies for low-value prescribing in secondary care: a systematic review

**DOI:** 10.1186/s43058-023-00498-0

**Published:** 2023-09-18

**Authors:** Jennifer Dunsmore, Eilidh Duncan, Sara MacLennan, James N’Dow, Steven MacLennan

**Affiliations:** 1 Academic Urology Unit, University of Aberdeen, Aberdeen, UK; 2 Health Service Research Unit, University of Aberdeen, Aberdeen, UK

**Keywords:** De-implementation, Strategies, Prescribing, Inappropriate, Low-value, Barriers, Facilitators, Consequences

## Abstract

**Background/aims:**

Considerable efforts have been made to improve guideline adherence in healthcare through de-implementation, such as decreasing the prescription of inappropriate medicines. However, we have limited knowledge about the effectiveness, barriers, facilitators and consequences of de-implementation strategies targeting inappropriate medication prescribing in secondary care settings. This review was conducted to understand these factors to contribute to better replication and optimisation of future de-implementation efforts to reduce low-value care.

**Methods:**

A systematic review of randomised control trials was conducted. Papers were identified through CINAHL, EMBASE, MEDLINE and Cochrane register of controlled trials to February 2021. Eligible studies were randomised control trials evaluating behavioural strategies to de-implement inappropriate prescribing in secondary healthcare. Risk of bias was assessed using the Cochrane Risk of Bias tool. Intervention characteristics, effectiveness, barriers, facilitators and consequences were identified in the study text and tabulated.

**Results:**

Eleven studies were included, of which seven were reported as effectively de-implementing low-value prescribing. Included studies were judged to be mainly at low to moderate risk for selection biases and generally high risk for performance and reporting biases. The majority of these strategies were clinical decision support at the ‘point of care’. Clinical decision support tools were the most common and effective. They were found to be a low-cost and simple strategy. However, barriers such as clinician’s reluctance to accept recommendations, or the clinical setting were potential barriers to their success. Educational strategies were the second most reported intervention type however the utility of educational strategies for de-implementation remains varied. Multiple barriers and facilitators relating to the environmental context, resources and knowledge were identified across studies as potentially influencing de-implementation. Various consequences were identified; however, few measured the impact of de-implementation on usual appropriate practice.

**Conclusion:**

This review offers insight into the intervention strategies, potential barriers, facilitators and consequences that may affect the de-implementation of low-value prescribing in secondary care. Identification of these key features helps understand how and why these strategies are effective and the wider (desirable or undesirable) impact of de-implementation. These findings can contribute to the successful replication or optimisation of strategies used to de-implement low-value prescribing practices in future.

**Trial registration:**

The review protocol was registered at PROSPERO (ID: CRD42021243944).

**Supplementary Information:**

The online version contains supplementary material available at 10.1186/s43058-023-00498-0.

Contributions to literature

- Overuse of healthcare is a global issue in which inappropriate prescribing is a key contributor.
- We explored intervention strategies, the barriers, facilitators and the consequences of hospital-based behavioural strategies aiming to de-implement low-value prescribing practices.
- Clinical decision support tools were common and effective in de-implementing low-value prescribing, whereas educational strategies reported mixed results.
- Barriers regarding the environment, context and clinician knowledge need to be considered in de-implementation efforts. Clinician beliefs and clinical settings need to be considered when using decision support strategies.
- These findings offer insight into the aspects that can contribute to successful replication and optimisation of de-implementation efforts for low-value prescribing in future.

## Background

Overuse of healthcare resources is a global issue and prescription of unnecessary medication is a key contributor to this [1]. Overprescribing of medication is defined by the UK Department of Health and Social care as medication that is not required or wanted, or where risk outweighs benefit [2]. Overprescribing is considered a ‘low-value’ practice, i.e. a practice that is not clinically [1] or cost-effective [3]. Despite efforts to encourage evidence-based practice and reduce low-value prescribing [4], healthcare professionals continue to prescribe unnecessary medication [5, 6]. To maintain a high standard of care, efficient use of resources, and to reduce potential harm to patients [1], it is important to eliminate low-value prescribing practices.

The process of eliminating ineffective or potentially harmful prescriptions can be conceptualised as ‘de-implementation’. De-implementation is not a well-defined concept and definitions vary between “abandonment of … practices” [7], or as any effort to “reduce” a practice or “address overuse” [8, 9]. In a scoping review, Niven and colleagues (2015), identified 43 terms in literature synonymous to de-implementation including “re-invest”, “discontinue” and “decline in use” [10]. Generally, these terms can fall into four types of de-implementation; removing, replacing, reducing or restricting a practice or treatment [11].

Although de-implementation has been conceptualised as the removal or reduction of a medical practice, it cannot be considered the opposite to implementation (i.e. the integration or increase) of a practice [7, 12]. The evidence base has highlighted that de-implementation is distinct from implementation [13–15]. Specific conceptual models for de-implementation have been developed to guide research [7, 10, 11, 16, 17] by highlighting areas for further exploration. Key features of barriers, facilitators, the type of target action, the intervention strategies and, the definition and measurement of outcomes of de-implementation have been highlighted as likely areas to differ from implementation. Further, the implications or consequences arising from achieving successful de-implementation outcomes were highlighted as another key area likely to differ from implementation [18].

Understanding the consequential effects of removing an established medical practice is crucial to evaluate de-implementation efforts [19, 20] and gain a comprehensive understanding of the full impact of de-implementation [18]. In a recent review of the consequences experienced in strategies used to reduce antibiotic prescribing, expected ‘trade-offs,’ such as an increased hospital stay, were viewed as a reasonable compromise for a successful de-implementation outcome [21]. However, unintended consequences, such as clinicians increasing their use of other medical practices to replace the practice being discontinued, may have possibly harmful repercussions [18], in addition to contradicting the process of de-implementation [21]. A fuller understanding of the nuanced impact of de-implementation alongside successful strategies and determinants will offer information to contribute to the growing evidence base which will aid better replication and optimisation of future de-implementation.

While implementation and de-implementation have been conceptualised as unique, implementation science offers a strong foundation to understand de-implementation [22, 23]. Existing implementation theory and methods have been utilised in de-implementation research considerably [24]. Frameworks such as the Theoretical Domains Framework (TDF) [25] have been applied to offer insight into de-implementation [26–29]. Most recently, the TDF has been successfully used in a multi-method study to identify barriers and facilitators of de-implementing of multiple low-value care practices in a critical care setting [30]. Parsons Leigh and colleagues successfully mapped findings from their systematic review and interview study to the TDF which provided a structured theoretical lens on important determinants of de-implementation in this setting. These existing approaches have also been used to understand the determinants of low-value prescribing behaviour, allow cumulative contributions to the expanding de-implementation evidence base and offer tangible information that can be used in future strategies [19].

Therefore, the aim of this study is to further understand the key features of de-implementation strategies aiming to reduce low-value prescribing. The insights of these efforts will contribute to better replication and optimisation of future de-implementation efforts. We used a systematic review approach to synthesise relevant literature.

### Review questions

This systematic review aimed to answer these questions:

1. What strategies have been used to de-implement low-value prescribing practices of secondary healthcare prescribers, and how effective were these strategies?
2. What are the reported barriers, facilitators and unintended consequences of these strategies?

## Review methods

### Design

#### Protocol registration

This systematic review is reported following the Preferred Reporting Items for Systematic reviews and Meta-Analyses (PRISMA) statement (Additional file 1). The protocol was registered at PROSPERO (ID: CRD42021243944).

#### Identification of studies

Studies were identified by searching CINAHL, EMBASE, MEDLINE and Cochrane register of controlled trials databases from inception to February 2021.

#### Search

A search strategy was developed with an Information Officer. The search was designed to identify randomised control trials (RCTs) evaluating behaviour change strategies to address a low-value or unnecessary medication prescriptions, within a secondary healthcare setting. De-implementation-related terms identified in a scoping review were the basis of this search strategy [10]. See the full search strategy in Table 1; the searches were adapted to the vocabulary and syntax of each database.

**Table 1:** Search strategy for MEDLINE

1. randomized controlled trial.pt
2. controlled clinical trial.pt
3. randomly.ab
4. trial.tw
5. (randomized or randomised).ab
6. placebo.ab
7. clinical trials as topic/
8. 1 or 2 or 3 or 4 or 5 or 6 or 7
9. (abandon* or delist* or de-list* or disinvest* or dis-invest* or discontinu* or dis-continu* or decommission* or de-commission* or deadopt* or de-adopt* or de-implement* or deimplement* or withdraw* or decreas* or re-invest* or reinvest* or declin* or chang* or realloc* or re-alloc*or remov* or replac* or stop* or reduc* or “do not do”).tw
10. ((inappropriate or unnecessary or excess* or ineffective or overus* or nonrecommended or unrecommended or “not recommended” or “low-value” or “low-value”) adj3 (prescrib$ or prescript$ or treatment? or care or practice? or service? or management)).tw
11. (behaviour change or behavioural change or behaviour* intervention? or behaviour* modification? or behaviour* strateg* or behaviour* techniques? or behaviour* tool? or behaviour* plan? or behaviour* program* or behavior change or behavioral change or behavior* intervention? or behavior* modification? or behavior* strateg* or behavior* technique? or behavior* tool? or behavior* plan? or behavior* program* or intervention? or strateg* or technique? or modification? or tool? or plan? or program*).tw
12. 9 and 10 and 11
13. (secondary care or hospital? or inpatient? or in-patient? or out-patient? or outpatient? or hospitalized or hospitalised or hospital patient).tw
14. exp Secondary care/
15. exp Hospitals/
16. 13 or 14 or 15
17. 8 and 12 and 16

#### Study selection (inclusion and exclusion criteria)

Inclusion and exclusion criteria were developed following the PICO (Population, Intervention, Context and Outcome) structure (Table 2). Eligible studies were RCTs comparing behavioural strategies to a control or other strategies. Studies had to include an intervention designed to reduce, remove, restrict or replace low-value medication prescription practice. Only studies reported in English were included. Studies had to target the initial prescribing part of the care process, rather than, at the time of review or reconciliation of medication. Prescribing here refers only to the prescription of medication, as opposed to prescribing or ordering tests or procedures. One author (JD) screened 100% of the title and abstracts, and 10% of these were independently double screened by a second author (SM). Double screening of titles and abstracts yielded a kappa score of 0.39, which is considered ‘fair’ agreement [31]. One author (JD) screened 100% of full-text articles, and 40% were independently double screened by a second author (SM).

**Table 2:** Inclusion and exclusion criteria

	**Inclusion**	**Exclusion**
**Study Design**	Randomised controlled trials	Non-randomised study design
**Population**	Prescribers in secondary care setting	Prescribers outside of secondary care prescribers e.g. primary care doctors
**Setting/ Context**	Secondary care	Community pharmacies GPs, care homes, palliative care, community care
**Target behaviour**	Prescribing therapeutic drugs	Screening, monitoring, surgery behaviours, prescriptions of supplements, prescriptions of other non-therapeutic drugs
**Intervention**	Any behavioural intervention used to de-implement low-value prescribing practices, including removal, reduction, replacement, or restriction of practice	Strategies not targeting prescribing behaviour, strategies aiming to increase prescribing rate, strategies targeting patients, strategies using diagnostic testing, use of tests to diagnose and strategies used in medical reviews
**Comparator(s)/ control**	Usual practice, another intervention or waitlist	No comparator group
**Primary Outcomes**	Change in prescribing including removal, reduction, replacement, or restriction of practice	Studies only reporting patient-related outcomes and not reporting clinical behaviour outcomes
**Secondary Outcomes**	Patient-related outcomes (e.g. disease recurrence, adverse events)	None
**Geographical Locations**	All	None
**Language**	English	Not English

#### Data extraction and coding

A bespoke data extraction form was developed and a priori headings were selected to gather data on the unique complexities of de-implementation guided by Norton and Chambers conceptual de-implementation framework [11, 18]. Headings included target actions, intervention strategies, barriers, facilitators, outcomes and consequences. Data was extracted verbatim where possible.

To allow comparisons, strategies were categorised using the Effective Practice and Organisation of Care Taxonomy (EPOC) [32]. The EPOC taxonomy offers a classification of health system interventions, including an overarching description category and subcategories of interventional strategies. All studies were allocated to overarching categories and relevant subcategories. Where appropriate, multifaceted strategies were categorised into multiple relevant subcategories.

Barriers and facilitators were the interpretation of the trial results as offered by the authors of the studies. Barriers were defined as any intrinsic or extrinsic influences faced by prescribers mentioned in the article text that may have hindered the de-implementation of low-value prescribing. Facilitators were defined as any intrinsic or extrinsic influences that may have enabled intervention success. Verbatim text of identified barriers and facilitators were summarised and mapped to the Theoretical Domains Framework (TDF) [25].

The TDF is a synthesis of 33 behavioural theories into 14 theoretical domains [25]: *Behavioural Regulation*, *Knowledge*, *Skills*, *Beliefs about Capabilities*, *Beliefs about Consequences*, *Intentions*, *Reinforcement*, *Goals*, *Memory*, *Attention and Decision Processes*, *Environmental Context and Resources*, *Social Influences*, *Optimism*, *Emotions*, and *Social/Professional Role and Identity*. The TDF is a theory-based tool that allows the categorisation of barriers and facilitators to understand key influences on a target behaviour.

Consequences were defined as repercussions arising from the implementation of the intervention. The diffusion of innovations literature provides a classification of consequences [33]. The framework suggested by Toma and colleagues was used to specify four classifications of consequences, including expected outcomes, which can be classed as either (1) desirable or (2) undesirable, and unexpected outcomes, which can also be (3) desirable or (4) undesirable [21]. Secondary outcomes, other planned or unplanned analyses and the study authors’ interpretations in the article text of potential consequences were extracted verbatim and categorised to these headings.

#### Data synthesis

We did not conduct a meta-analysis due to study heterogeneity in several factors of the included studies including variety in intervention strategies, the target illness and medication. Data was tabulated into evidence tables: (1) characteristics of included studies; (2) strategies within each study classified by the EPOC taxonomy; (3) reported intervention descriptions and their effects; (4) identified barriers to de-implementation categorised to the TDF; (6) identified facilitators of de-implementation categorised to the TDF; (7) identified consequences of de-implementation specified with Toma and colleagues framework.

#### Quality assessment

The risk of bias was assessed by one reviewer (JD) and a 10% sample was independently assessed by a second reviewer (SM). The tool recommended for assessing the risk of bias in RCTs outlined in the Cochrane Handbook for Systematic Reviews of Strategies [34] was used. Risk of bias arising from the randomisation process, timing of recruitment of participants (in cluster-randomised trials), deviations from the intended interventions, missing outcome data, measurement of the outcome and selection of the reported result were assessed. Disagreements were resolved by discussion within our research team.

## Results of the review

### Details of included and excluded studies

**Fig. 1:**
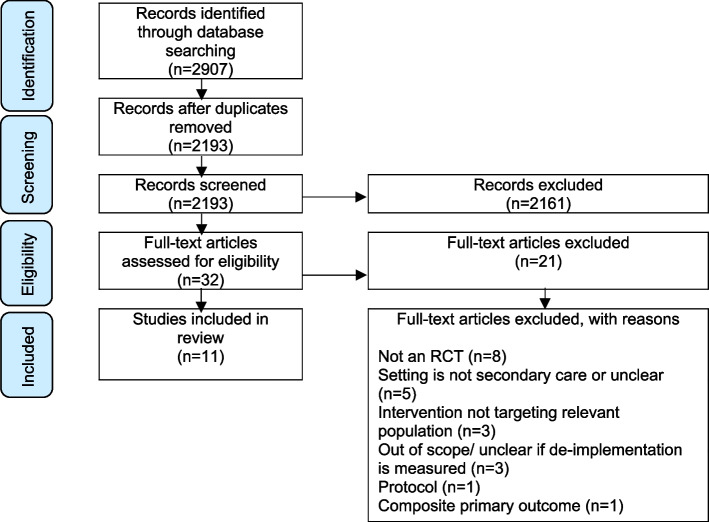
PRISMA flow diagram

The database search returned 2907 titles and abstracts. Following the removal of duplicates, 2193 records remained. Thirty-two full-text articles were screened for eligibility. Eleven studies were included in this review. See the PRISMA flow diagram in Fig. 1. Main reasons for exclusion at the full text reviewing stage, included non-RCT study designs, the setting of the study was not in secondary care, and where it was unclear if the study had a de-implementation focus.

### Included study characteristics

Study characteristics for the 11 included studies can be found in Table 3. Eight studies compared their strategies to a usual care comparator group [35–42] and three studies offered a partial or adapted intervention [43–45] as their comparator. Seven studies were cluster RCTs [36, 37, 39, 43–45], including one stepped wedge [42]. Two interventions included strategies targeting both the healthcare professional and the patient [37, 45]. See additional file 2 for verbatim intervention and comparison arm names, descriptions and counts.

**Table 3:** Characteristics of included studies

Author and year of Study	Type of low-value care prescribing	Type of trial	Country	Clinical setting	Patient type	Type of de-implementation
**Daley et al., 2018** [35]	Antibiotics for asymptomatic bacteriuria	Intervention vs control	Canada	Acute care hospitals	Adults	“Reduce”
**Franchi et al., 2016** [43]	Drug prescription in elderly patients	Intervention vs active control	Italy	Geriatric or internal medical wards	Elderly patients	“Reduce”
**Menya et al., 2015** [36]	Artemisinin-based combination therapies for suspected malaria	Intervention vs control	Kenya	Health centres	Children and adults	“Reduce”
**Metlay et al., 2007** [37]	Antibiotic use for acute respiratory infections	Intervention vs control	USA	Hospital emergency departments	Adults	“Reduce”
**Moja et al., 2019** [38]	Prescription medications	Intervention vs control	Italy	Internal medicine wards of 1 hospital	Adults and children	“Reduce”
**Opondo et al., 2011** [44]	Antibiotic use in non-bloody diarrhoea	Intervention vs active control	Kenya	District hospitals	Children	“Reduce”
**Paul et al., 2006** [39]	Empirical antibiotic treatment	Intervention vs control	Israel, Italy and Germany	Various wards	Adults	Unclear
**Terrell et al., 2009** [40]	Potentially inappropriate medications in older adults	Intervention vs control	USA	Emergency department	Elderly patients	“Reduce”
**Terrell et al., 2010** [41]	Excessive medication dosing for patients in renal impairment	Intervention vs control	USA	Academic emergency department	Adults	“Reduce”
**van de Maat et al., 2020** [42]	Antibiotic prescription in children with suspected lower respiratory tract infection	Intervention vs control	The Netherlands	Emergency departments and Urgent Care clinics	Children	“Reduce”
**Yadav et al., 2019** [45]	Antibiotic prescribing for Acute respiratory infection	Intervention vs active control	USA	Emergency departments and Urgent Care clinics	Adults and children	“Reduce”

The type of de-implementation as “reducing” low-value prescribing was identified in all studies except one. The majority of studies were conducted in high-income countries [35, 37–43, 45], and two took place in Kenya [36, 44]. Studies were set in emergency departments or urgent care units [37, 40–42, 45], other ward types [38, 39, 43] or whole hospitals [35, 36, 44]. The type of low-value prescribing being targeted tended to be inappropriate antibiotics for a range of illnesses [35, 37, 39, 42, 44, 45]; however, general inappropriate drug prescriptions, especially in the elderly, were also reported [40, 43].

The 11 studies reported 11 intervention arms. Table 4 shows classifications of intervention arms to the Effective Practice and Organisation of Care (EPOC) Taxonomy [32]. The overarching categories of ‘Implementation Strategies, Interventions targeted at healthcare workers’ and ‘Financial Arrangements, Targeted financial incentives for health professionals and healthcare organisations’ were identified. Four interventions were multi-faceted with between 4 and 8 subcategories identified. ‘Educational materials’ and ‘Audit and Feedback’ were the most used strategies in these multifaceted interventions [37, 43–45]. Seven interventions were single strategy, six were described as ‘reminders’ [35, 38–42] and one study was classified as ‘pay for performance’ [36]. Reminders were the most commonly used strategies (*n* = 6), educational strategies including educational meetings and educational materials were the second most common (*n* = 4).

**Table 4:** Classifications of included de-implementation interventions to the Effective Practice and Organisation of Care Taxonomy (EPOC) categories and subcategories

	**Daley et al., 2018** [35]	**Franchi et al., 2016** [43]	**Menya et al., 2015 **[36]	**Metlay et al., 2007 **[37]	**Moja et al., 2019 **[38]	**Opondo et al., 2011** [44]	**Paul et al., 2006 **[39]	**Terrell et al., 2009 **[40]	**Terrell et al., 2010 **[41]	**van de Maat et al., 2020 **[42]	**Yadav et al., 2019 **[45]	**Number of studies using each strategy**
**Implementation strategies, interventions targeted at healthcare workers and subcategories**												
**Reminders**	✓	✓			✓		✓	✓	✓	✓	✓	8
**Educational materials**		✓		✓		✓					✓	4
**Audit and feedback**				✓		✓					✓	3
**Educational meetings**				✓							✓	2
**Monitoring performance of delivery of healthcare**						✓					✓	2
**Local opinion leaders**						✓					✓	2
**Patient-mediated interventions**				✓							✓	2
**Inter-professional educational**						✓						1
**Clinical Practice Guidelines**						✓						1
**Managerial supervision**						✓						1
**Local consensus processes**											✓	1
**Financial arrangements, targeted financial incentives for health professionals and healthcare organisations and subcategories**												
**Pay for performance**			✓									1
**Number of strategies identified in each study**	1	2	1	4	1	7	1	1	1	1	8	

Studies with an active comparator used adapted versions of the strategies employed in the intervention arm, for example, Franchie and colleagues provided educational materials in both arms, but limited the control arm to selected modules [43]. 

### Quality assessment of studies

**Fig. 2:**
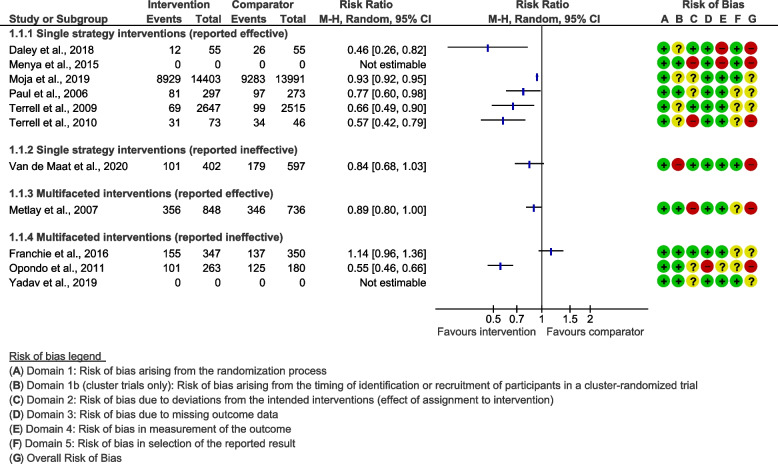
Forest plot illustrating raw data and unadjusted differences

Risk of bias results for studies can be seen in Fig. 2. Six studies were considered to have an overall high risk of bias [35–37, 41, 42, 44] and five had a moderate risk of bias [38–40, 43, 45]. No studies were found to have an overall low risk of bias. Generally, studies tended to perform well on biases relating to the randomisation process and missing data management. However, deviating from the intended intervention domain presented the most risk of bias, as three studies had high [36, 37, 41] and four moderate risk [38, 39, 44, 45] for this domain. It should be noted that those assigned high risk for this domain tended to suffer risk of bias due to participants being aware of their participation in the trial or the allocation of the intervention, rather than deviations from intervention strategies that may have arisen from the context. Six studies were assessed as having a moderate risk of bias in the domain of selection of reported results [37, 39–41, 43, 44], which was mainly due to the majority of studies not referring to a published protocol.

### Intervention effectiveness

The main aims of the included studies were to assess the effectiveness of de-implementation strategies (e.g. evaluate the effectiveness of a clinical decision support system to reduce inappropriate antibiotic prescribing in children admitted to hospital). Table 5 shows the intervention type and where studies were found effective. Seven studies reported strategies being effective in reducing low-value prescribing [35–41] of these, four studies were of high risk of bias [35–37, 41] and three were moderate risk [38–40]. One study reported weak evidence of an intervention effect, after adjusting for patient, clinician level factors and study period [44]. The remaining three strategies did not show any differences across arms in de-implementing inappropriate prescribing [42, 43, 45]. Additional file 3 shows the verbatim outcome definitions and results for each study.

**Table 5:** Intervention description, type and reported effectiveness

**Study**	**Verbatim intervention description** (Page number and section)	**Type of intervention**	**Reported as effective**
**Daley et al., 2018** [35]	“modified reporting of positive urine cultures” (p814, Introduction)	Single	Yes
**Metlay et al., 2007 **[37]	“multidimensional educational intervention” (p223, Interventions)	Multi-faceted	Yes
**Moja et al., 2019 **[38]	“Medilogy Decision Support System” (p3, Development of CDSS)	Single	Yes
**Paul et al., 2006 **[39]	“computerized decision support system for antibiotic treatment” (p1238, Abstract)	Single	Yes
**Terrell et al., 2009 **[40]	“computer assisted decision support” (p1388, Abstract)	Single	Yes
**Terrell et al., 2010 **[41]	“decision support in a computerized physician order entry system” (p623, Abstract)	Single	Yes
**Menya et al., 2015 **[36]	“facility-directed, performance based incentives” (p4, Incentive Intervention)	Single	Yes
**Franchi et al., 2016 **[43]	“e-learning program teaching CGA [Comprehensive Geriatric Assessment] and basic geriatric pharmacological notions” (p53, Abstract)	Multi-faceted	No
**Opondo et al., 2011 **[44]	“multi-faceted quality improvement intervention” (p1, Title)	Multi-faceted	No
**van de Maat et al., 2020 **[42]	“a validated clinical prediction model (Feverkidstool) was implemented as a decision rule guiding antibiotic prescription.” (p6, Intervention)	Single	No
**Yadav et al., 2019 **[45]	“stewardship intervention that additionally incorporates behavioral nudges” (p719, Abstract)	Multi-faceted	No

The most reported effective strategies were classed as ‘reminders’, offered at the point of care [35, 38–42]. Reminders are defined by the EPOC Taxonomy [32] as any intervention that prompts action, such as decision support aids. Although in varying formats, ‘reminders’ were single-strategy clinical decision support systems. These decision support systems varied as some offered thresholds to guide appropriate prescribing decisions [42], accounted for or restricted additional diagnostic information [35, 39] or provided recommendations of appropriate substitutes [38, 40, 41]. These strategies were offered in emergency and non-emergency settings; however, the use of clinical decision support systems was exclusive to high-income countries. Educational strategies, including educational materials and meetings, were the second most common. However, only one of four studies using educational strategies was found to be effective [37].

Eight studies reported outcomes as ‘inappropriate’ prescribing behaviour (i.e. measured the number of unnecessary prescriptions) and three reported outcomes as ‘appropriate’ (i.e. measured the amount of necessary prescriptions), data and calculated odds ratios are presented in the forest plot in Fig. 2 for comparison. Where data was not available or easily extracted, study authors were contacted. Menya et al. (2015) and Yadav et al. (2019) were unable to offer complete data.

### Barriers and facilitators of de-implementation

#### Barriers

Only one study proactively identified potential barriers, of alert fatigue and unimportant reminders, to inform intervention design [38]. Two other studies quantitatively measured potential sources of barriers, including a real-time assessment of reasons for rejecting prescription recommendations [40], and a survey of clinicians’ attitudes toward antibiotic resistance before and after the intervention implementation [45]. In other studies, authors interpretations referred to potential barriers but there was no formal measurement or indication if these potential barriers were considered in intervention design. Barriers and their related Theoretical Domains Framework (TDF) domains are reported in Table 6. More detailed summaries of identified barriers and related TDF domains can be found in Additional file 4.

**Table 6:** Categorisation of barriers to the Theoretical Domain Framework (TDF) domains

Study	Intervention strategies (EPOC categories)	Reported as effective	Environmental context and resources	Knowledge	Social influences	Behavioural regulation	Skills	Goals	Memory, attention and decision processes	Social/professional role and identity	Reinforcement	Optimism	Emotions	Beliefs about capabilities	Beliefs about consequences	Intentions	Number of TDF domains identified in each study
**Daley et al., 2018** [35]	Reminders	Yes	-	-	-	-	-	-	-	-	-	-	-	-	-	-	0
**Metlay et al., 2007 **[37]	Educational meetings, Educational materials, Audit and feedback, Patient-mediated interventions	Yes	✓	✓													2
**Moja et al., 2019 **[38]	Reminders	Yes	✓						✓								2
**Paul et al., 2006 **[39]	Reminders	Yes	✓	✓													2
**Terrell et al., 2009 **[40]	Reminders	Yes	✓		✓	✓				✓							4
**Terrell et al., 2010 **[41]	Reminders	Yes	✓	✓	✓												3
**Menya et al., 2015 **[36]	Pay for performance	Yes	✓														1
**Franchi et al., 2016 **[43]	Educational materials, Reminders	No	✓	✓	✓			✓									4
**Opondo et al., 2011 **[44]	Inter-professional education, Clinical Practice Guidelines, Educational materials, Monitoring performance of delivery of healthcare, Managerial supervision, Local opinion leaders, Audit and feedback	No	✓	✓			✓										3
**van de Maat et al., 2020 **[42]	Reminders	No	✓														1
**Yadav et al., 2019 **[45]	Educational meetings, Reminders, Educational materials, Patient-mediated interventions, Local consensus processes, Local opinion leaders, Monitoring the performance of the delivery of healthcare, Audit and Feedback	No	-	-	-	-	-	-	-	-	-	-	-	-	-	-	0
**Number of studies with barriers associated with each TDF domain**			9	5	3	1	1	1	1	1	0	0	0	0	0	0	

The most frequently identified barriers related to the *Environmental Context and Resources* domain (*n* = 9). Issues including; an unestablished electronic health record [38, 43], emergency settings experiencing issues of continuity of care and severely ill patients [37], a lack of morale in healthcare staff and an increased volume of patients [36] were highlighted as potential barriers to de-implementation. The need for additional information [42] or data entry required [38] for the decision support strategies to work were also reported as barriers. Additionally, the type of information offered and the timing of the decision support intervention being too late [38] were also identified.

Barriers relating to the *Knowledge* domain were the second most frequently identified (*n* = 5). Clinicians knowledge of; what constitutes an inappropriate prescription [37], the illnesses that are responsive to antibiotics [37], where a reduction in dose is required [41] and how to utilise an intervention were reported as potential barriers [36]. In two of the studies aiming to reduce antibiotic prescribing, authors reported that intervention information that contradicted clinicians training [44] or treatment decisions [39] was likely to prevent successful de-implementation. In addition, although not widely mentioned, *Goal*-related barriers such as a clinician’s lack of interest or underestimation of low-value prescription issues [43] may also have the potential to impact on de-implementation.

*Social influences* barriers were also cited to potentially impact de-implementation (*n* = 3). Patients were reported to demand medication [40] or have a general influence on prescriptions [43]. Social influence barriers also included clinicians attempting to be consistent with colleague’s prescriptions [40, 41] but were only cited in studies conducted in emergency settings. The specific culture of emergency settings was noted to have an impact on de-implementation. Clinicians in these contexts were ensuring the patient gets the medication they are expecting and not disrupting another prescriber’s decision. These contextual barriers were reported to impact new and refilled prescriptions [40, 41].

#### Facilitators

Most facilitators identified (Table 7) related to the *Environmental context and resources* domain (*n* = 8). Ensuring the intervention was easy to use [35, 38], tailored to the local context [39, 45], supported by infrastructure [36, 40, 45], available at the time of a prescription decision [40] and of low cost [35, 39–41] were identified as likely to facilitate de-implementation. More detailed summaries of identified barriers and related TDF domains can be found in Additional file 5.

**Table 7:** Categorisation of facilitators to the Theoretical Domain Framework (TDF) domains

Study	Intervention description	Reported as effective	Environmental context and resources	Knowledge	Social influences	Behavioural regulation	Skills	Goals	Memory, attention and decision processes	Social/professional role and identity	Reinforcement	Optimism	Emotions	Beliefs about capabilities	Beliefs about consequences	Intentions	Number of TDF domains identified in studies
**Daley et al., 2018** [35]	Reminders	Yes	✓														1
**Metlay et al., 2007 **[37]	Educational meetings, Educational materials, Audit and feedback, Patient-mediated interventions	Yes	-	-	-	-	-	-	-	-	-	-	-	-	-	-	0
**Moja et al., 2019 **[38]	Reminders	Yes	✓						✓								2
**Paul et al., 2006 **[39]	Reminders	Yes	✓														1
**Terrell et al., 2009 **[40]	Reminders	Yes	✓				✓		✓								3
**Terrell et al., 2010 **[41]	Reminders	Yes	✓														1
**Menya et al., 2015 **[36]	Pay for performance	Yes	✓	✓	✓												3
**Franchi et al., 2016 **[43]	Educational materials, Reminders	No		✓													1
**Opondo et al., 2011 **[44]	Inter-professional education, Clinical Practice Guidelines, Educational materials, Monitoring performance of delivery of healthcare, Managerial supervision, Local opinion leaders, Audit and feedback	No	✓				✓										2
**van de Maat et al., 2020 **[42]	Reminders	No	-	-	-	-	-	-	-	-	-	-	-	-	-	-	0
**Yadav et al., 2019 **[45]	Educational meetings, Reminders, Educational materials, Patient-mediated interventions, Local consensus processes, Local opinion leaders, Monitoring the performance of the delivery of healthcare, Audit and Feedback	No	✓		✓			✓		✓					✓		5
**Number of studies with facilitators associated with each TDF domain**			8	2	2	0	2	1	2	1	0	0	0	0	1	0	

Other possible facilitators of de-implementation included clinicians having a correct understanding of appropriate prescriptions (*Knowledge*) [36, 43], obtaining a fuller record of symptoms (*Skills*) [38], being more experienced (*Skills*) [40], being provided with a choice in treatment options (*Social/Professional role and Identity*) [45] and avoiding alert fatigue (*Memory, Attention and Decision Processes*) [38]. Healthcare staff feeling that changing their behaviour can have a positive impact on patients (*Beliefs about consequences*), and motivation to further reduce already low rates of inappropriate prescribing (*Goals*) [45] were identified as other possible facilitators.

### Consequences of de-implementation

Consequences that could have been caused by the intervention were identified in nine studies [35–40, 42, 43, 45] and were identified through secondary outcomes and additional analyses. Table 8 provides the number of identified consequences categorised to the conceptual framework [21]. Desirable (*n* = 22), undesirable (*n* = 4), expected (*n* = 6) and unexpected consequences (n = 4) were identified. Many authors of included studies did not clarify if a difference (or non-difference) between arms indicated a desirable or undesirable outcome and even less so if these outcomes were expected or unexpected. More details of consequences can be found in Additional file 6.

**Table 8:** Number of consequences identified in studies

	**Number of consequences identified**	**Number of studies reporting consequences (of 11 studies)**
Desirable	22	8
Undesirable	4	3
Expected	6	4
Unexpected	4	3
Unclear	20	8

Desirable and undesirable consequences were usually related to patient safety. Desirable consequences included a reduction in duration of fever [39], fever at day 7 and secondary antibiotic prescriptions [42] and reduced length of stay in hospital [35, 39]; however, one study reported this could have been a cofounded finding [35]. Conversely, an intervention using educational materials (and a decision support system, which was not utilised) found patients at intervention sites had a significantly longer length of stay [43] but did not make clear if this was unexpected. Three studies reported a desirable consequence that their intervention did not adversely impact the appropriate process of care or prescriptions being given to patients who required such medications [36, 37, 42].

One study, using reminders, reported a financially desirable consequence, where a change in the type of medication used was associated with a reduction in medication cost [39], whereas, another intervention using modified lab reporting was not associated with a cost saving [35].

Other undesirable consequences were identified. Consequences arising from the clinician’s interaction with the intervention were also identified. One study using a decision support system reported that prescribing outcomes improved for all types of reminders a clinician received (i.e. reminders of evidence-based practice or potential drug interactions) and did not increase alert fatigue, which are desirable consequences [38]. However, the amount of time required to select a medication using the decision support increased over the course of the trial, again, Moja and colleagues did not define this as unexpected or a reasonable trade-off [38].

In one of the multifaceted interventions, consequences were identified through a pre-post measure of attitudes towards antibiotic stewardship [45]. More clinicians reported a positive attitude and agreed that stewardship was important following the intervention, which could indicate a desirable consequence. However, in the same study, clinicians also continued to feel that a stewardship intervention would not impact on their decision-making in future [45], which could be an undesirable consequence as it may be counterproductive in de-implementation efforts.

Other consequences could refer to the level of use or acceptance of an intervention. A high level of compliance or use of the intervention is assumed to correspond with intervention success; however generally, a potential trade-off or realistic expectation is that the intervention will not reach 100% uptake. For example, one study reported 43% of recommendations were accepted and continued to produce an effective outcome; however, it was not clear if this level of acceptance was expected or desired [40].

## Discussion

This review aimed to understand the effectiveness, barriers, facilitators, and consequences of strategies de-implementing low-value medication prescribing in secondary healthcare. The majority of included studies were found to significantly reduce inappropriate medication prescribing (*n* = 7/11). Included studies addressed various low-value prescribing practices, the majority addressed antibiotic use (*n* = 6) and others targeted malaria therapies in non-malaria patients, unnecessary medication for older people or in general. We deductively extracted data to further understand the complexities of de-implementation as conceptualised in Norton and Chambers de-implementation framework [18].

Despite the widespread efforts to reduce low-value care [46], only a few strategies were identified and even fewer successfully de-implemented low-value prescribing practice in secondary healthcare. Effective strategies included single strategy clinician decision support systems (*n* = 6), financial incentives (*n* = 1) and a multi-faceted intervention including components of education and audit and feedback (*n* = 1). This is consistent with the literature as strategies to reduce low-value care across the health system found clinicians’ decision support and multifaceted interventions (usually including education) to be most utilised and effective [8].

In our review, clinical decision support systems were the most common and effective strategy to de-implement inappropriate prescribing. This aligns with a more recent systematic review of strategies that addressed low-value care in cancer services [47]. Alishahi Tabriz and colleagues postulated that the success of decision support strategies was due to aids being ‘active’ as opposed to ‘passive’. ‘Active’ strategies are intentional and exerted efforts to facilitate change in behaviour or ways of working [47]. ‘Active’ strategies have previously been found useful in de-implementation. In a scoping review conducted in 2015 of de-implementation interventions in healthcare more generally, de-implementation happened when evidence was diffused; however, those using an ‘active’ intervention to aid the use of evidence, such as clinician education or withdrawing a medication from the market, were more likely to lead to successful de-implementation [10]. To ensure effective de-implementation, efforts should move away from passive dissemination of evidence and offer ‘active’, intentional and exerted interventions, such as clinical decision support systems, to facilitate de-implementation.

Educational strategies, of educational materials and educational meetings, were the second most commonly used strategies to reduce low-value prescribing, however only one reported a significant effect [37]. These educational strategies were multifaceted and were usually paired with audit and feedback or monitoring. Previous literature has shown educational strategies to be effective when paired with other strategies [8]. Additionally, multi-faceted strategies using clinical educational or academic detailing among other strategies are likely to facilitate de-implementation and curb low-value care compared to being used alone [48]. Metlay and colleagues, authors of the effective multifaceted intervention, using education, audit and feedback, did not offer reasons for success; however, they noted the need to distinguish the most suitable ‘active ingredient/s’ of their intervention [37]. There have been strides to identify the active ingredients or behavioural change techniques used in de-implementation interventions however, more needs to be done to understand the utility of these components and which ingredients would facilitate the most significant effect [29]. Where educational strategies are paired with other strategies, a clearer understanding of the degree to which education content, or the behaviour change techniques within educational strategies can facilitate de-implementation is required to optimise their contribution to de-implementation in future.

Consideration of determinants that may prevent or enable de-implementation [49] were also explored in our review. Only one study in our review explicitly identified potential barriers before implementing their intervention [38], others offered interpretations of potentially influential determinants retrospectively. Most identified potential barriers related to the TDF domain of *Environmental context and resources*, particularly strategies utilising decision support. Intervening at the point of care was seen as beneficial [41] and can disrupt clinicians’ decision-making to influence a change in decision [50], but is likely to face many pitfalls [51]. Only one study, although effective, found prescribing decisions may have been made in advance of interacting with computer systems and decision support may have been offered “too late” [38]. Across included studies decision support was usually offered at ‘point of care’ (we assume once a prescription was decided). If the decision process for prescribing happens before the time a clinician engages with a computer, a change in the decision and subsequent de-implementation of the selected behaviour may be more difficult or unsustained. Explicit specification of ‘when’ and ‘where’ these decision aid strategies operate are required to understand the extent these context factors affect the decision support success [52]. Additionally, measurement of these factors will allow for further optimisation of ‘point of care’ de-implementation strategies.

The setting and culture in which an intervention is embedded should be a main consideration for designing and optimising de-implementation strategies [20]. Barriers relating to the wider context (as highlighted in the *Environmental context and resources* domain) were frequently identified across studies. Two of five studies conducted in emergency settings, referred to the ‘specific culture’ of these settings hindering de-implementation [37, 40]. Continuity of care, dealing with very sickly patients and the desire to not deviate from other clinician’s plans were viewed as potential barriers in these settings. The importance of the setting of the de-implementation intervention has been emphasised in previous research. In a systematic review of determinants of low-value care nursing practices, characteristics of the setting such as the type of department, organisational norms and structure were found to be influential in both, the use of low-value practices and de-implementation process [53]. Additionally, the wider culture including political support or pressure and economy were also notable determinants of de-implementation [53], although these factors were not identified in the current review. Intervention designers are required to have a clear appreciation of the context and setting within which de-implementation efforts are implemented to better understand and attempt to influence these determinants.

Consequences produced by the intervention, where reported, tended to be ‘desirable’ (i.e. reduced length of stay). Consequences were classified to a framework as suggested by Toma and colleagues [21] where phrasing of findings or authors’ interpretations made classification possible. It was difficult to understand if a measured outcome not having a difference between arms (e.g. no difference between arms for frequency of return hospital visits) should be categorised as a ‘desirable’ or an ‘undesirable’ consequence. However, where a difference was experienced such as a significant reduction in the length of stay in hospital in intervention arms [35, 39], classification was more easily allocated. Clearer expectations of potential consequences are required to understand if undesirable or unexpected consequences are acceptable trade-offs of de-implementation.

Consideration of how the reduction or removal of a low-value practice may impact appropriate care has already been highlighted as a potential consequence that should be captured in any de-implementation effort [19]. Only three (of 11) studies in the current review measured the impact of their strategies on appropriate prescriptions. Evaluations of strategies need to widen the scope, to consider expected, unexpected, desirable and undesirable consequences of de-implementation on patients, providers and organisations to account for any impact on appropriate care caused by the de-implementation of low-value care [20]. Further research on the impact of and recurrence of these consequences will contribute to a fuller understanding of the impact of de-implementation strategies on appropriate and necessary care [21].

The continuation of low-value prescribing practices is an evidence-practice gap which requires a change in clinical behaviour. This review considered the determinants, strategies and consequences that influence de-implementation. This review has highlighted the relevant ‘complexities’ using Norton and Chamber’s framework [18] to further understand the process of de-implementation. To ensure effective de-implementation in future, strategies need to be informed by formulation work to understand determinants and account for potential consequences. This can be achieved through rigorous and theoretically informed research which in turn will contribute to an accumulation of knowledge about how and why de-implementation strategies work [54].

### Strengths and limitations

The strength of this review was its comprehensive search strategy. Developed with an information officer, the search strategy included the terms unique to the field of de-implementation [10] which ensured relevant studies were identified. Another strength was the comprehensive use of conceptual frameworks to understand the features of de-implementation and key areas required for further understanding of de-implementation. Comparisons across frameworks provide a theoretical lens and help identify where there are similarities or where gaps in knowledge exist.

However, there were limitations. First, only studies written in English, with a RCT design were included. Other study designs may have provided more information about barriers and facilitators or consequences of de-implementation strategies. Second, studies were excluded if they failed to define the setting or where the intervention target behaviour was not clearly defined as de-implementation. Unspecific language such as ‘change’ or ‘improve’ often used to describe de-implementation strategies was not captured in our search strategy. This meant that studies with an aim to reduce inappropriate practices may have been excluded on this basis.

A final limitation is that a meta-analysis was not performed to assess the effectiveness of included de-implementation strategies; instead, this review reported if they were effective or not. This review only included RCTs, considered the gold standard for the evaluation of strategies, as opposed to uncontrolled studies that may be poorer in quality. However, all included RCT risk of biases were assessed as high and moderate; therefore, quality should be a consideration in the interpretation of these results.

## Conclusions

In conclusion, this review has demonstrated that multiple intervention strategies were found to effectively de-implement inappropriate prescribing in secondary healthcare. Clinical decision support systems were the most effective and educational strategies may be useful; however, more research needs to be done to establish the degree to which these components are effective. Generally, environmental, contextual, social and knowledge-driven barriers and facilitators need to be taken into consideration when replicating or optimising de-implementation strategies. Specification of features, such as timing and context, should also be reported to gain insight to how best to optimise these strategies in future. Finally, any expected, unexpected or desirable and undesirable consequences caused by the intervention need to be measured to ensure the accumulation of knowledge of possible consequences to fully account for the impact of de-implementation.

### Supplementary Information

**Additional file 1. **PRISMA Checklist for systematic review and Abstracts.

**Additional file 2. **Table of Number of types of comparators and Table of Verbatim Intervention and comparator Name and Descriptions.

**Additional file 3. **Table of Outcomes and effects of the intervention.

**Additional file 4. **Table of Barriers to de-implementation categorised to the TDF Domains.

**Additional file 5. **Table of Facilitators to de-implementation categorised to the TDF Domains.

**Additional file 6. **Table of Reported consequences in included studies.

## Data Availability

The data generated or analysed in this review is available from the corresponding author on reasonable request.

## Abbreviations

RCT: Randomised control trial
RoB: Risk of bias
TDF: Theoretical Domains Framework
EPOC: Effective Practice and Organisation of Care

## References

[1] Brownlee S, Chalkidou K, Doust J, Elshaug AG, Glasziou P, Heath I (2017). Evidence for overuse of medical services around the world. Lancet.

[2] Department of Health & Social Care. Good for you, good for us, good for everybody: a plan to reduce overprescribing to make patient care better and safer, support the NHS, and reduce carbon emissions. 2021;85.

[3] NICE. Our principles | Who we are | About [Internet]. NICE. NICE; 2022 [cited 2022 Aug 9]. Available from: https://www.nice.org.uk/about/who-we-are/our-principles.

[4] Choosing Wisely UK. About Choosing Wisely UK [Internet]. Choosing Wisely UK. 2022. Cited 2022 June 10. Available from: https://www.choosingwisely.co.uk/about-choosing-wisely-uk/.

[5] Rosenberg A, Agiro A, Gottlieb M, Barron J, Brady P, Liu Y (2015). Early Trends Among Seven Recommendations From the Choosing Wisely Campaign. JAMA Intern Med.

[6] Rourke EJ (2022). Ten Years of Choosing Wisely to Reduce Low-Value Care. N Engl J Med.

[7] Prasad V, Ioannidis JP (2014). Evidence-based de-implementation for contradicted, unproven, and aspiring healthcare practices. Implement Sci.

[8] Colla CH, Mainor AJ, Hargreaves C, Sequist T, Morden N (2017). Interventions Aimed at Reducing Use of Low-Value Health Services: A Systematic Review. Med Care Res Rev.

[9] Ellen ME, Wilson MG, Vélez M, Shach R, Lavis JN, Grimshaw JM (2018). Addressing overuse of health services in health systems: a critical interpretive synthesis. Health Res Policy Syst.

[10] Niven DJ, Mrklas KJ, Holodinsky JK, Straus SE, Hemmelgarn BR, Jeffs LP (2015). Towards understanding the de-adoption of low-value clinical practices: a scoping review. BMC Med.

[11] Norton WE, Chambers DA, Kramer BS (2019). Conceptualizing De-Implementation in Cancer Care Delivery. J Clin Oncol.

[12] van Bodegom-Vos L, Davidoff F, Marang-van de Mheen PJ (2017). Implementation and de-implementation: two sides of the same coin?. BMJ Qual Saf.

[13] Patey AM, Grimshaw JM, Francis JJ (2021). Changing behaviour, ‘more or less’: do implementation and de-implementation interventions include different behaviour change techniques?. Implement Sci.

[14] Prusaczyk B, Swindle T, Curran G (2020). Defining and conceptualizing outcomes for de-implementation: key distinctions from implementation outcomes. Implementation Sci Commun.

[15] Patey AM, Hurt CS, Grimshaw JM, Francis JJ (2018). Changing behaviour ‘more or less’—do theories of behaviour inform strategies for implementation and de-implementation? A critical interpretive synthesis. Implementation Sci.

[16] Parsons Leigh J, Niven DJ, Boyd JM, Stelfox HT. Developing a framework to guide the de-adoption of low-value clinical practices in acute care medicine: a study protocol. BMC Health Serv Res. 2017;17. Cited 2022 May 6. 10.1186/s12913-017-2005-x. 10.1186/s12913-017-2005-x PMC5247804 28103931

[17] Powell AA, Bloomfield HE, Burgess DJ, Wilt TJ, Partin MR (2013). A Conceptual Framework for Understanding and Reducing Overuse by Primary Care Providers. Med Care Res Rev.

[18] Norton WE, Chambers DA (2020). Unpacking the complexities of de-implementing inappropriate health interventions. Implement Sci.

[19] Kerr EA, Kullgren JT, Saini SD (2017). Choosing Wisely: How To Fulfill The Promise In The Next 5 Years. Health Aff.

[20] Mafi JN, Parchman M (2018). Low-value care: an intractable global problem with no quick fix. BMJ Qual Saf.

[21] Toma M, Davey PG, Marwick CA, Guthrie B (2017). A framework for ensuring a balanced accounting of the impact of antimicrobial stewardship interventions. J Antimicrob Chemother.

[22] Walsh-Bailey C, Tsai E, Tabak RG, Morshed AB, Norton WE, McKay VR (2021). A scoping review of de-implementation frameworks and models. Implement Sci.

[23] Grimshaw JM, Patey AM, Kirkham KR, Hall A, Dowling SK, Rodondi N (2020). De-implementing wisely: developing the evidence base to reduce low-value care. BMJ Qual Saf.

[24] Nilsen P, Ingvarsson S, Hasson H, von Thiele SU, Augustsson H (2020). Theories, models, and frameworks for de-implementation of low-value care: A scoping review of the literature. Implementation Res Pract.

[25] Cane J, O’Connor D, Michie S (2012). Validation of the theoretical domains framework for use in behaviour change and implementation research. Implement Sci.

[26] Voorn VMA, Wentink MM, Kaptein AA, Koopman-van Gemert AWMM, So-Osman C, Marang-van de Mheen PJ (2014). Perceived barriers among physicians for stopping non-cost-effective blood-saving measures in total hip and total knee arthroplasties. Transfusion.

[27] Cullinan S, Fleming A, O’Mahony D, Ryan C, O’Sullivan D, Gallagher P (2015). Doctors’ perspectives on the barriers to appropriate prescribing in older hospitalized patients: a qualitative study. Br J Clin Pharmacol.

[28] Skolarus TA, Forman J, Sparks JB, Metreger T, Hawley ST, Caram MV (2021). Learning from the “tail end” of de-implementation: the case of chemical castration for localized prostate cancer. Implementation Science Communications.

[29] Patey AM, Islam R, Francis JJ, Bryson GL, Grimshaw JM, the Canada PRIME Plus Team (2012). Anesthesiologists’ and surgeons’ perceptions about routine pre-operative testing in low-risk patients: application of the Theoretical Domains Framework (TDF) to identify factors that influence physicians’ decisions to order pre-operative tests. Implement Sci.

[30] Parsons Leigh J, Sypes EE, Straus SE, Demiantschuk D, Ma H, Brundin-Mather R (2022). Determinants of the de-implementation of low-value care: a multi-method study. BMC Health Serv Res.

[31] Landis JR, Koch GG (1977). The Measurement of Observer Agreement for Categorical Data. Biometrics.

[32] Effective Practice and Organisation of Care. Effective Practice and Organisation of Care (EPOC). EPOC Taxonomy. 2015. Cited 2021 June 16. Available from: https://epoc.cochrane.org/sites/epoc.cochrane.org/files/public/uploads/taxonomy/epoc_taxonomy.pdf.

[33] Rogers EM (2003). Diffusion of Innovations.

[34] Higgins JPT, Thomas J, Chandler J, Cumpston M, Li T, Page MJ, et al. Cochrane Handbook for Systematic Reviews of Interventions. Wiley; 2019. Available from: https://training.cochrane.org/handbook. 10.1002/14651858.ED000142 PMC10284251 31643080

[35] Daley P, Garcia D, Inayatullah R, Penney C, Boyd S (2018). Modified Reporting of Positive Urine Cultures to Reduce Inappropriate Treatment of Asymptomatic Bacteriuria Among Nonpregnant, Noncatheterized Inpatients: A Randomized Controlled Trial. Infect Control Hosp Epidemiol.

[36] Menya D, Platt A, Manji I, Sang E, Wafula R, Ren J (2015). Using pay for performance incentives (P4P) to improve management of suspected malaria fevers in rural Kenya: a cluster randomized controlled trial. BMC Med.

[37] Metlay JP, Camargo CA, MacKenzie T, McCulloch C, Maselli J, Levin SK (2007). Cluster-Randomized Trial to Improve Antibiotic Use for Adults With Acute Respiratory Infections Treated in Emergency Departments. Ann Emerg Med.

[38] Moja L, Polo Friz H, Capobussi M, Kwag K, Banzi R, Ruggiero F (2019). Effectiveness of a Hospital-Based Computerized Decision Support System on Clinician Recommendations and Patient Outcomes: A Randomized Clinical Trial. JAMA Netw Open..

[39] Paul M, Andreassen S, Tacconelli E, Nielsen AD, Almanasreh N, Frank U (2006). Improving empirical antibiotic treatment using TREAT, a computerized decision support system: cluster randomized trial. J Antimicrob Chemother.

[40] Terrell KM, Perkins AJ, Dexter PR, Hui SL, Callahan CM, Miller DK (2009). Computerized Decision Support to Reduce Potentially Inappropriate Prescribing to Older Emergency Department Patients: A Randomized, Controlled Trial: Decision support for inappropriate prescribing. J Am Geriatr Soc.

[41] Terrell KM, Perkins AJ, Hui SL, Callahan CM, Dexter PR, Miller DK (2010). Computerized Decision Support for Medication Dosing in Renal Insufficiency: A Randomized Controlled Trial. Ann Emerg Med.

[42] van de Maat JS, Peeters D, Nieboer D, van Wermeskerken A-M, Smit FJ, Noordzij JG (2020). Evaluation of a clinical decision rule to guide antibiotic prescription in children with suspected lower respiratory tract infection in The Netherlands: A stepped-wedge cluster randomised trial. PLoS Med..

[43] Franchi C, Tettamanti M, Djade CD, Pasina L, Mannucci PM, Onder G (2016). E-learning in order to improve drug prescription for hospitalized older patients: a cluster-randomized controlled study: E-learning to improve drug prescription. Br J Clin Pharmacol.

[44] Opondo C, Ayieko P, Ntoburi S, Wagai J, Opiyo N, Irimu G (2011). Effect of a multi-faceted quality improvement intervention on inappropriate antibiotic use in children with non-bloody diarrhoea admitted to district hospitals in Kenya. BMC Pediatr.

[45] Yadav K, Meeker D, Mistry RD, Doctor JN, Fleming-Dutra KE, Fleischman RJ (2019). A Multifaceted Intervention Improves Prescribing for Acute Respiratory Infection for Adults and Children in Emergency Department and Urgent Care Settings. Choo EK, editor. Acad Emerg Med..

[46] Levinson W, Kallewaard M, Bhatia RS, Wolfson D, Shortt S, Kerr EA (2015). “Choosing Wisely”: a growing international campaign. BMJ Qual Saf.

[47] Alishahi Tabriz A, Turner K, Clary A, Hong Y-R, Nguyen OT, Wei G (2022). De-implementing low-value care in cancer care delivery: a systematic review. Implement Sci.

[48] Cliff BQ, Avanceña ALv, Hirth RA, Lee S-YD (2021). The Impact of Choosing Wisely Interventions on Low-Value Medical Services: A Systematic Review. Milbank Quart.

[49] van Bodegom-Vos L, Davidoff F, de Mheen PJM (2017). Implementation and de-implementation: two sides of the same coin?. BMJ Qual Saf.

[50] Helfrich CD, Rose AJ, Hartmann CW, van Bodegom-Vos L, Graham ID, Wood SJ (2018). How the dual process model of human cognition can inform efforts to de-implement ineffective and harmful clinical practices: A preliminary model of unlearning and substitution. J Eval Clin Pract.

[51] Sutton RT, Pincock D, Baumgart DC, Sadowski DC, Fedorak RN, Kroeker KI (2020). An overview of clinical decision support systems: benefits, risks, and strategies for success. Npj Digital Medicine..

[52] Proctor EK, Powell BJ, McMillen JC (2013). Implementation strategies: recommendations for specifying and reporting. Implement Sci.

[53] Augustsson H, Ingvarsson S, Nilsen P, von Thiele SU, Muli I, Dervish J (2021). Determinants for the use and de-implementation of low-value care in health care: a scoping review. Implementation Sci Commun.

[54] Lewis CC, Boyd MR, Walsh-Bailey C, Lyon AR, Beidas R, Mittman B (2020). A systematic review of empirical studies examining mechanisms of implementation in health. Implement Sci.

