# Supplementary material for: Effectiveness of de-implementation strategies for low-value prescribing in secondary care: a systematic review
Source: Implement Sci Commun. 2023 Sep 18;4:115. doi: 10.1186/s43058-023-00498-0 (PMC10507868; doi:10.1186/s43058-023-00498-0)
Supplement: Supplementary file 2 — Additional file 2. Table of Number of types of comparators and Table of Verbatim Intervention and comparator Name and Descriptions. [file 43058_2023_498_MOESM2_ESM.docx]

| **Table of Number of types of comparators** | | |
| --- | --- | --- |
|  | Usual Practice comparator | ‘Active’ comparator |
| Number of studies | 8 | 3 |

| **Table of Verbatim Intervention and comparator Name and Descriptions** | | |
| --- | --- | --- |
| **Study** | **Verbatim Intervention Name (Page number and section)** | **Verbatim Intervention Description (Page number and section)** |
| **Daley et al., 2018** | "modified reporting of positive urine cultures" (p. 814, Introduction) | "The modified report informed the physician that significant bacterial growth was detected, but bacterial identification and susceptibility information were withheld unless requested. Specimens randomized to the modified report were reported as: “This POSITIVE urine culture may represent asymptomatic bacteriuria or urinary tract infection. If urinary tract infection is suspected clinically, please call the microbiology laboratory at [phone] between 0900 to 2300, or the microbiology technologist on-call at [phone] at night, for identification and susceptibility results.”" (p. 815, Intervention) |
| **Franchi et al., 2016** | "e-learning program teaching CGA [Comprehensive Geriatric Assessment] and basic geriatric pharmacological notions" (p. 53, abstract) | "E-learning platform. E-learning was delivered through an interactive web-based platform accessed by a personal identification code and password. A system meant to assess the implementation and completion by clinicians of the elearning training was also set up. An automatic electronic system recorded and rated for each participating clinician the number of access occasions, the time spent on each elearning module, the right/wrong answers and the number of attempts needed to complete each module correctly… delivered to clinicians on the wards randomly assigned to the intervention arm included notions of CGA and geriatric pharmacology, together with training for the use of a third generation assessment instrument (InterRAI Acute Care) [20, 21]. The course on geriatric pharmacology was structured in three main areas and five modules as follows: Area 1: main concepts of CGA (Module A). Area 2: general geriatric pharmacology notions (Module B). Area 3: prescription appropriateness and related issues in older adults: (a) assessment and management of patients exposed to polypharmacy (Module C); (b) criteria and tools for the revision and evaluation of prescription appropriateness in older people, such as Beers Criteria, Screening Tool of Older Person’s Prescriptions (STOPP), Assessing Care of the Vulnerable Elderly (ACOVE), Inappropriate Prescribing in the Elderly Tool (IPET) and the Medication Appropriateness Index (MAI) (Module D); (c) criteria and tools to evaluate potential drug–drug interactions (Module E). The access to and utilization of each teaching module was linked to a self evaluation test and to specific centralized controls. Each module was divided in four sub-modules that each participant completed with specific case reports and questions. The INTERcheck® software, a computerized prescription support system [22], was made available to clinicians in the intervention arm through the interactive web-based platform, separately from the electronic clinical report form." (p. 55, Intervention) |
| **Franchi et al., 2016**  (Comparator) | "e-learning for the control arm" (P. 55, Intervention) | "The e-learning program for clinicians of the control arm consisted only of refresher on the basic notions of geriatric pharmacology using Module B as a weapon." (p. 55, Intervention) |
| **Menya et al., 2015** | "facility-directed, performance based incentives" (p. 4, Incentive Intervention) | "Facilities in the intervention arm received incentive payments based on seven performance indicators…The incentives were designed to foster cooperation between departments (pharmacy, laboratory and clinical care) and none were punitive. Spending of the incentive funds was subject to certain restrictions according to the requirements of the government’s HSSF: they could only be used by the facility for equipment, supplies, repairs and basic labor [29]; and not for payments to individual clinicians or employees." (p. 4, Incentive Intervention) |
| **Metlay et al., 2007** | "multidimensional educational intervention" (p. 223, Interventions) | "The multidimensional educational intervention emphasized principles of judicious antibiotic use for acute respiratory tract infections5,15,16 and was targeted at clinicians and patients. We based our intervention on the Predisposing, Reinforcing and Enabling Constructs in Educational Diagnosis and Evaluation (PRECEDE) model of behavior change.17 This model emphasizes the inclusion of predisposing strategies (i.e., increasing provider knowledge about acute respiratory tract infection management), reinforcing strategies (i.e., delivering feedback to providers on past patterns of antibiotic prescribing), and enabling strategies (patient education to reduce antibiotic demand)…  [1]…Clinical leaders [who lead training] … were provided with an informational slide set and reprints of relevant supporting journal articles from emergency medicine journals…  [2]…The slide set emphasized the evidence against efficacy of antibiotic therapy for upper respiratory tract infection and acute bronchitis that were based on the Centers for Disease Control and Prevention’s (CDC’s) “Principles of Appropriate Antibiotic Use for Adults With Acute Respiratory Tract Infections”5 (available online at <http://medicine.ucsf.edu/impaact/clinic.html)…>  [3]…Sites were provided with aggregated site-specific data on their use of antibiotics for acute respiratory tract infections in the preintervention winter year (measured as described below). These results were benchmarked against the average for all other VA or non-VA sites (as appropriate), as well as against an evidence-based benchmark, according to literature review.18" (p. 223, Interventions) |
| **Metlay et al., 2007**  (separate patient intervention) | "patient educational intervention" (p. 223 | "Waiting room posters [1] and brochures [2] incorporated messages that are part of the CDC’s Get Smart antibiotics campaign (available online at http://www.cdc.gov/drugresistance/community/campaign_info.htm). In addition, a computerized educational module housed on a video kiosk [3] in the waiting room provided information tailored to specific acute respiratory tract infection symptoms... Examination room posters summarized clinical trial evidence emphasizing the lack of clinical benefit for patients with acute bronchitis treated with antibiotics, as well as issues related to antibiotic resistance" (p. 223, Interventions) |
| **Moja et al., 2019** | "Medilogy Decision Support System (MediDSS)." (p. 3, Development of CDSS) | "patient-specific guidance and reminders, including therapeutic suggestions, EBMEDS integrates structured patient data from EHRs using the following controlled vocabularies and code sets: medical history, vital signs, diagnoses (International Classification of Diseases, Ninth Revision), symptoms (International Classification of Primary Care, Second Edition), medications (World Health Organization Anatomical Therapeutic Chemical Classification System), and immunization dates, allergies, and laboratory data (Logical Observation Identifiers Names and Codes), as well as imaging reports. Reminders were automatically generated and presented on screen when clinicians entered new information or accessed a patient’s EHR. We endeavored to integrate the CDSS into the clinical workflow by implementing alert features to grade different alerting priorities (e.g., using color codes). However, we preferred noninterruptive reminders" (p. 3, CDSS and EHR Integration) |
| **Opondo et al., 2011** | "multi-faceted quality improvement intervention" (p. 1, Title) | "The full intervention had several components including:…  [1] the five-and-a-half day theory and practical training (ETAT+) in recommended practices for 30-34 hospital staff at each intervention site (in total 90 nurses, 11 medical officers and 29 clinical officers providing paediatric care referred to here as ‘clinicians’ were trained across the four hospitals)…  [2] clinical practice guidelines…  [3] job-aids for all health workers providing paediatric care…  [4] 2-3 monthly supervision of hospitals’ implementation by a paediatrician from the study team to discuss progress in guideline implementation, informed by data from surveys when available (see below), and…  [5] identify local strategies for problem-solving with senior hospital staff (described by Nzinga et al. [23])…  [6] a local facilitator from among the hospital staff assigned to promote guideline implementation for 18 months…  [7] six-monthly surveys to assess progress and collect data for the study; and written and face-to-face feedback on findings after each survey. Written and face to face feedback focused on key trial outcomes but also provided information on inappropriate antibiotic use for non-bloody diarrhoea amongst many other indicators of quality of care. Face to face feedback was provided by those undertaking supervision six monthly, however the wider supervisory process (2-3monthly) was not standardised tending to focus on key, hospital specific problems related to pre-identified key indicators (18) and follow up of locally developed action plans" (p. 3, The intervention) |
| **Opondo et al., 2011**  (Comparator) | "partial intervention" (p. 3, The intervention) | "A partial intervention was delivered to ‘control’ hospitals including: the same [1] 1.5 days didactic training on the use of guidelines as in the full 5.5 days course that was given to 35-40 hospital staff at each site (107 nurses, 6 medical officers and 21 clinical officers in total across the four sites)…  [2] the clinical practice guidelines and…  [3] job-aids; and…  [4] written feedback after contemporaneous surveys" (p. 3, The intervention) |
| **Paul et al., 2006** | "computerized decision support system for antibiotic treatment (TREAT)" (p. 1238, Abstract) | "TREAT recommends treatment by highlighting the three top-rank antibiotic regimens, with the highest cost-benefit difference, including no antibiotic treatment" (P. 1239, Methods) |
| **Terrell et al., 2009** | "computer assisted decision support" (p. 1388, Abstract) | "They limited the recommended medications to those on the formulary at Wishard. The right column of the supplemental table lists the therapies that were suggested in place of each inappropriate medication. For most, the recommendations varied according to the indication for prescribing the medication. Some medications had specific doses or durations of therapy recommended" (p. 1389, Randomisation and Intervention) |
| **Terrell et al., 2010** | "decision support in a computerized physician order entry system" (p. 623, Abstract) | "The intervention was computerized decision support designed to facilitate the appropriate dosing of medications for adult patients (i.e., patients aged 18 years and older) with renal insufficiency who were being discharged home from the ED...The decision support system estimated creatinine clearance level with the Cockcroft-Gault equation...Decision support was provided when an intervention physician prescribed a targeted medication to a patient whose estimated creatinine clearance level was below the threshold for dosage adjustment for that particular medication" (p. 625, Intervention) |
| **van de Maat et al., 2020** | "phase, a validated clinical prediction model (Feverkidstool) was implemented as a decision rule guiding antibiotic prescription" (p. 6, Intervention) | "predefined decision thresholds that would guide antibiotic treatment decisions, balancing positive and negative likelihood ratios and the consequences of over- and undertreatment" (p. 6, Intervention) |
| **Yadav et al., 2019**  (Inclusion of patient intervention) | "stewardship intervention that additionally incorporates behavioral nudges (enhanced intervention)." (p. 719, Abstract) | [1] “Provider education; Educational presentations, electronic reminders of ARI guidelines, Get Smart brochures” [2] “Patient education; CDC Get Smart posters in waiting rooms, discharge handouts”  [3] “Provider commitment-enhanced patient education; Personalized posters in examination rooms including modified Get Smart content directed at patients, enhanced with clinicians’ photos and signed public commitment to antibiotic stewardship” [4] “Physician champion: Designated physician at each site who will lead provider education and be an advocate for antibiotic stewardship” [5] “Departmental feedback: Monthly aggregate of antibiotic prescribing practices for ARI from EHR data provided to departmental leadership” [6] “Peer comparison: Personalized monthly performance ranking delivered by e-mail with each physician receiving designation of being a “top performer” (top decile) or “not a top performer” for avoiding antibiotics for ARI.” (p. 722, Methods, Table 1) |
| **Yadav et al., 2019**  (Comparator)  (Inclusion of patient intervention) | "Adapted Intervention" (p. 720, Methods) Stewardship intervention without behavioural nudges) | [1] “Provider education; Educational presentations, electronic reminders of ARI guidelines, Get Smart brochures” [2] “Patient education; CDC Get Smart posters in waiting rooms, discharge handouts”  [3] “Physician champion: Designated physician at each site who will lead provider education and be an advocate for antibiotic stewardship” [4] “Departmental feedback: Monthly aggregate of antibiotic prescribing practices for ARI from EHR data provided to departmental leadership” (p. 722, Methods, Table 1) |
